# Behavioural Treatments for Tourette Syndrome: An Evidence-Based Review

**DOI:** 10.3233/BEN-120309

**Published:** 2013

**Authors:** Madeleine Frank, Andrea Eugenio Cavanna

**Affiliations:** ^1^The Michael Trimble Neuropsychiatry Research GroupDepartment of NeuropsychiatryUniversity of Birmingham and BSMHFTBirminghamUK; ^2^College of Medical and Dental SciencesUniversity of BirminghamBirminghamUK; ^3^Sobell Department of Motor Neuroscience and Movement DisordersInstitute of NeurologyUCLLondonUK

**Keywords:** Tourette syndrome, behavioural treatments, habit reversal training, massed negative practice, supportive psychotherapy, exposure with response prevention

## Abstract

Tourette syndrome (TS) is a disorder characterised by multiple motor and vocal tics and is frequently associated with behavioural problems. Tics are known to be affected by internal factors such as inner tension and external factors such as the surrounding environment. A number of behavioural treatments have been suggested to treat the symptoms of TS, in addition to pharmacotherapy and surgery for the most severe cases. This review compiled all the studies investigating behavioural therapies for TS, briefly describing each technique and assessing the evidence in order to determine which of these appear to be effective. Different behavioural therapies that were used included habit reversal training (HRT), massed negative practice, supportive psychotherapy, exposure with response prevention, self-monitoring, cognitive-behavioural therapy, relaxation therapy, assertiveness training, contingency management, a tension-reduction technique and biofeedback training. Overall, HRT is the best-studied and most widely-used technique and there is sufficient experimental evidence to suggest that it is an effective treatment. Most of the other treatments, however, require further investigation to evaluate their efficacy. Specifically, evidence suggests that exposure with response prevention and self-monitoring are effective, and more research is needed to determine the therapeutic value of the other treatments. As most of the studies investigating behavioural treatments for TS are small-sample or single-case studies, larger randomised controlled trials are advocated.

## 1. Introduction

Tourette syndrome (TS) is a neurodevelopmental disorder characterised by the chronic presence of multiple motor and phonic tics, i.e. involuntary movements and utterances [1,2]. A recent review of the existing epidemiological studies for TS established that TS is relatively common, affecting around 1% of school-age children [3]. Tics vary between patients in terms of their anatomic location, frequency and severity, and these factors themselves change over time within the individual [4]. Co-morbid behavioural problems are reported by about 90% of patients with TS [5]. The most common psychiatric co-morbidities include obsessive compulsive disorder (OCD) [6], attention deficit hyperactivity disorder (ADHD) [7], affective disorders [8], impulse control disorders [9] and personality disorders [10]. Neuroimaging studies of subjects with TS have frequently implicated the basal ganglia and associated cortices with the disorder, demonstrating reduced activity within this area relative to control subjects [11]. Findings suggest that the basal ganglia portions of the striatum and its dopaminergic circuitry may be the key areas involved in the pathophysiology of TS, as this is the region regulating motor and behavioural expression [12]. As a result of this neurobiological model of TS, pharmacotherapy is currently

ISSN 0953-4180/13/$27.50 © 2013 – IOS Press and the authors. All rights reserved the commonest treatment [13], although it tends not to be used in patients with mild symptoms [14]. A behavioural model of TS however acknowledges that tics are susceptible to being affected by environmental events [15] and can to some extent be controlled by the individual [16]. Thereby behavioural interventions can also be implemented, especially when medications are found to be ineffective or have intolerable side-effects [13]. These treatments aim to teach patients strategies to consciously modify their behaviour to reduce the severity of their tics. Ideally, treatment of severe TS should include a combination of psychological and pharmacological therapies [14]. This review explores the different behavioural approaches used in TS and reflects on their relative effectiveness.

## 2. Methods

A comprehensive electronic literature search was conducted for this review, in line with the PRISMA guidelines for systematic literature reviews [17]. The databases accessed included Pubmed, Ovid, Medline, PsycInfo and Google Scholar. The following search terms were used: "Tourette syndrome", "tics", "behavioural treatment", "habit reversal training", "massed negative practice", "supportive psychotherapy", "exposure with response prevention", "self-monitoring", "cognitive-behavioural therapy", "relaxation therapy", "assertiveness training", "contingency management" and "biofeedback training". The different therapies were found mainly from reviews evaluating behavioural treatments for TS, or they were brought up by the search engines. Reference lists of pertinent articles were also used to find related studies. Finally, the tables of contents of the journals which had published the most literature on the topic were reviewed. These included Behaviour Research and Therapy, the Journal of Applied Behavior Analysis, Behavior Modification, and the Journal of Behavior Therapy and Experimental Psychiatry.

## 3. Results

Our systematic literature search found the following behavioural treatments for TS: HRT, massed negative practice (MNP), supportive psychotherapy (SP), exposure with response prevention (ERP), self-monitoring (SM), cognitive-behavioural therapy (CBT), relaxation therapy (RT), assertiveness training (AT), contingency management (CM), a tension-reduction technique and biofeedback training (BT). Table 1 summarises the results of the studies included in this review.

### *3.1. Habit reversal training (HRT)*

HRT is the most commonly used behavioural treatment for TS, as several studies have proven its clinical efficacy in reducing tics [18]. HRT is a multicomponent treatment, the main stages of which are recording, awareness training, competing response practice, habit control motivation and generalisation training [19]. In order to prevent a tic from occurring, the patient is taught how to recognise warning signs such as premonitory urges, and to perform a specific competing response using antagonistic muscles to the muscles required to perform the tic [19]. Since its original conception, HRT has become simplified and can usually be taught in eight 1-hour sessions or less, with no loss of effectiveness [20]. To date, eight randomised controlled trials (RCTs) [21–28] and 14 single-case studies [19,29–41] have been conducted investigating the efficacy of HRT. Carr and Chong [18] assessed 12 studies investigating the use of HRT to treat tics. 94% of the participants observed tic reductions, most of which maintained at long-term follow-up assessment, thus leading to the conclusion that HRT is an effective treatment for tics. When compared with other behavioural treatments, HRT has consistently been found to be of equal or superior efficacy [13]. The recently published large RCTs by Piacentini et al. [27] and Wilhelm et al. [28] alleviated much of the concern regarding the widespread use of small-n designs or group designs with small samples. The first study was conducted in the United States and included 126 children and adolescents with moderate to severe TS, who were randomly assigned to undergo a comprehensive behavioural intervention for tics (CBIT: a combination of tic awareness and HRT) or to be part of the control group who received supportive psychotherapy (SP) and education sessions. The CBIT treatment and the control treatment were delivered in 8 sessions across 10 weeks. The authors reported that about 53% of the children who received CBIT were judged significantly improved compared with 19% of those who did not receive CBIT. Specifically, CBIT gave a significantly greater decrease in tic severity and improved the psychological, social and school functioning for children and adolescents compared to the control group. These effects were maintained after a 6-month follow up for 87% of the children who had initially been responsive to the treatment. The second study adopted a similar paradigm, with slightly less positive results, in an overall adult sample of 122 patients.

Table 1
Summary of studies using behavioural techniques to treat tic symptoms

| Study | Behavioural technique(s) | No. participants | Age range or mean age (years) | Diagnosis | Type of study | Results |
|---|---|---|---|---|---|---|
| Azrin and Nunn 1973 | HRT | 12 | 5–64 | Tic disorder | Single-case studies | Tics were reduced by 99% after 3 weeks of HRT. This was maintained at 7-month follow-up. |
| Finney et al. 1983 | HRT | 2 | 11–12 | Tic disorder | Single-case studies | Tics were reduced to low levels at the end of treatment, and this was maintained at 12-month follow-up. |
| Azrin and Peterson 1988 | HRT | 3 | 28–42 | TS | Single-case studies | HRT reduced tic severity by 64–99% at home and by 93–95% in the clinic. |
| Sharenow et al. 1989 | HRT | 3 | 32–66 | Tic disorder | Single-case studies | HRT produced significant reduction in tic expression in all subjects. This was maintained at 1-year follow-up in 2 patients (the 3rd was lost to follow-up). |
| Woods et al. 1996 | HRT | 4 | 8–12 | Tic disorders, TS, ADHD | Single-case studies | Tic frequency decreased in all participants. This was maintained at follow-up (10 to 17 weeks post-treatment) (except for one participant who was lost to follow-up). |
| Azrin and Peterson 1990 | HRT | 10 | 6–36 | TS | Single-case studies | Tics were reduced by 93% at the end of HRT treatment. |
| Carr and Bailey 1996 | HRT | 1 | 9 | TS | Single-case study | Tics were reduced by almost 70%. This was maintained at 1-month follow-up. |
| Carr et al. 1996 | HRT | 2 | 12 | TS | Single-case studies | No change in tic expression was found, however it turned out that the participants were not carrying out the treatment as instructed. |
| Clarke et al. 2001 | HRT | 4 | 11–16 | TS | Single-case studies | 3 of the 4 participants showed significant decreases in tic frequency, and 1 participant showed mild decreases, within a school setting. These results were maintained at 10-week follow-up. |
| Woods et al. 2003 | HRT | 5 | 10–13 | TS | Single-case studies | Vocal tics were reduced by 82% overall, and there was no increase in motor tics. These results were maintained in 3 participants at 3-month follow-up. |
| Miltenberger et al. 1985 | HRT vs. simplified HRT | $N = 4$ (HRT) vs. $n = 5$ (simplified HRT) | 9–60 | Tic disorder | RCT | Tic expression was significantly reduced in all subjects receiving simplified HRT, and in 3 of 4 subjects receiving full HRT. |
| Azrin et al. 1980 | HRT vs. MNP | $N = 10$ (HRT) vs. $n = 12$ (MNP) | 16–62 | Tic disorder | RCT | 18-month follow-up showed a 97% reduction in tics for the HRT group vs. a 30% reduction for the MNP group. |
| Wilhelm et al. 2003 | HRT vs. SP | $N = 16$ (HRT) vs. $n = 13$ (SP) | 36.2 (HRT) vs. 33.2 (SP) | TS | RCT | Tic severity decreased significantly in the HRT group. This was maintained at 10-month follow-up. SP was not found to reduce tic severity. |
| Deckersbach et al. 2006 | HRT vs. SP | $N = 15$ (HRT) vs. $n = 15$ (SP) | 36.6 (HRT) vs. 33.6 (SP) | TS | RCT | Both treatments improved life-satisfaction and psychosocial functioning at 6-month follow-up. HRT alone reduced tic severity. |

Table 1, continued

| Study | Behavioural technique(s) | No. participants | Age range or mean age (years) | Diagnosis | Type of study | Results |
|---|---|---|---|---|---|---|
| Piacentini et al. 2010 | HRT vs. SP | $N = 61$ (HRT) vs. $n = 65$ (SP) | 11.6 (HRT) vs. 11.7 (SP) | TS or chronic tic disorder | RCT | Tics were significantly improved in 53% of the patients who received HRT vs. 19% of those who received SP. These effects were maintained at 6-month follow-up for 87% of the patients who had initially been responsive. |
| Wilhelm et al. 2012 | HRT vs. SP | $N = 63$ (HRT) vs. $n = 59$ (SP) | 31.6 (HRT) vs. 31.5 (SP) | TS or chronic tic disorder | RCT | Tics were significantly improved in 38% of the patients who received HRT vs. 6% of those who received SP. These effects were maintained at 6-month follow-up for 80% of the patients who were available for follow-up. |
| Verdellen et al. 2004 | ERP vs. HRT | $N = 21$ (ERP) vs. $n = 22$ (HRT) | 22 (ERP) vs. 19.2 (HRT) | TS (6 with OCD and 13 with ADHD) | RCT | Both groups showed reductions in YGTSS and tic frequency at 3-month follow-up. There was no significant difference between groups. |
| O'Connor et al. 1997 | HRT vs. CBT | $N = 7$ (HRT) vs. $n = 7$ (BCT) | 23–49 | Tic disorder | RCT | At 3-month follow-up, both groups showed reduced tic frequency, increased control over the tic and lower EMG level. There was no significant difference between groups. |
| Ollendick 1981 | SM and HRT | 2 | 9–11 | Tic disorder | Single-case studies | SM (SM plus HRT in one case) reduced tic frequency to near-zero. This was maintained at 1-year follow-up. |
| Peterson and Azrin 1992 | HRT, SM and RT | 6 | 10–40 | TS | Single-case studies | Tics were reduced by 55% with HRT, 44% with SM and 32% wit RT. These reductions are significant from baseline, but none of the treatments are significantly different from the others. |
| Woods and Twohig 2002 | HRT and CM | 3 | 7-16 | Tic disorder | Single-case studies | Tic frequency decreased to low levels in 2 participants and this was maintained at 3-month follow-up. One participant's tics did not improve due to non-compliance. CM was added to the treatment but this was ineffective. |
| Feldman and Werry 1966 | MNP | 1 | 13 | Tic disorder | Single-case study | Tic frequency increased dramatically during treatment period and decreased to baseline levels after therapy. |
| Nicassio et al. 1972 | MNP | 2 | 22–33 | Tic disorder | Single-case studies | One patient's tics were eliminated and this was maintained at 18-month follow-up. The second patient's tics did not change significantly. |
| Knepler and Sewall 1974 | MNP | 1 | 20 | Tic disorder | Single-case study | 53% reduction in tics at baseline, 95% reduction at 3 month follow-up, > 98% reduction at 6-month follow-up. |
| Lahey et al. 1973 | MNP and CM | 1 | 10 | TS | Single-case study | MNP decreased tic frequency by half, and CM decreased tic frequency even more. These results did not seem sustainable following treatment. |

Table 1, continued

| Study | Behavioural technique(s) | No. participants | Age range or mean age (years) | Diagnosis | Type of study | Results |
|---|---|---|---|---|---|---|
| Frederick 1971 | MNP and RT | 1 | Middle-aged | Hysterical neurosis associated with tics and blepharospasm | Single-case study | Treatment eliminated the tics completely. This was maintained at 9-month follow-up. |
| Canavan and Powell 1981 | MNP and RT | 1 | 24 | TS | Single-case study | MNP was found to be ineffective and even increase tic frequency. RT failed to produce any significant long-term reduction in tic frequency. |
| Turpin and Powell 1984 | MNP and RT | 3 | 27–36 | TS | Single-case studies | Treatments provided no long-term tic reduction, except RT which produced a moderate decrease in tic frequency in one patient. |
| Crawley and Powell 1986 | MNP and RT | 1 | 53 | Tic disorder | Single-case study | Tic frequency increased during MNP and RT. |
| St James-Roberts and Powell 1979 | MNP vs. RT | 1 | 45 | Tic disorder | Single-case study | MNP was found to be significantly more effective than RT at decreasing tic frequency. |
| Tophoff 1973 | MNP, RT and AT | 1 | 13 | TS | Single-case study | Complete disappearance of tics, maintained at 4-month follow-up. |
| Hoogduin et al. 1997 | ERP | 4 | 12–30 | TS | Single-case studies | ERP caused tic reduction at follow-up in 3 patients (15%–99%) but one patient relapsed completely at 2-month follow-up after baseline improvement of 60%. |
| Meidinger et al. 2005 | ERP | 6 | 7–20 | TS | Single-case studies | There was no rebound effect on tic frequency following ERP sessions. |
| Wetterneck and Woods 2006 | ERP | 1 | 11 | TS | Single-case study | Treatment reduced repetitive behaviours but effects were not maintained at 3-month follow-up. |
| Verdellen et al. 2007 | ERP | 20 | 22.4 | TS | Prospective study | Prolonged tic suppression in ERP is not followed by an increase of tics to release tension. |
| Verdellen et al. 2008 | ERP | 19 | 7–55 | TS | Prospective study | Decrease in severity of the premonitory sensation before a tic was associated with a decrease in tic frequency. |
| Thomas et al. 1971 | SM | 1 | 18 | TS | Single-case study | SM was found to reduce vocal tics dramatically, and to reduce motor tics. |
| Hutzell et al. 1974 | SM | 1 | 11 | TS | Single-case study | Tic frequency was reduced at 1-year follow-up. |
| Billings 1978 | SM | 1 | 17 | Tic disorder | Single-case study | SM was found to reduce, although not eliminate, frequency and intensity of tics at 6-month follow-up. |
| Wright and Miltenberger 1987 | SM | 1 | 19 | Tic disorder | Single-case study | Tic frequency decreased to an extent that they were no longer very noticeable to other people. This was maintained at 1-, 2-, 4- and 8-month follow-up. |
| Savicki and Carlin 1972 | SM and CM | 1 | 17 | TS | Single-case study | Vocal tics were reduced to an acceptable level during treatment, but returned at high level at 10-week follow-up, after which treatment was discontinued. |

Table 1, continued

| Study | Behavioural technique(s) | No. participants | Age range or mean age (years) | Diagnosis | Type of study | Results |
|---|---|---|---|---|---|---|
| Varni et al. 1978 | SM and CM | 1 | 7 | Tic disorder, LD and hyperactivity | Single-case study | Tics were eliminated. This was maintained at 32-week follow-up. |
| O'Connor et al. 2009 | CBT | $N = 23$ (medication) vs. $n = 53$ (no medication) | 43.2 (medication) and 40.5 (no medication) | TS and other tic disorders | Controlled trial | CBT was found to improve clinical variables (tic severity and frequency, anxiety, depression) in both medicated and unmedicated groups. There was no significant difference in outcome between the two groups. |
| O'Connor et al. 2001 | CBT vs. waitlist | $N = 52$ (CBT) vs. 38 (waitlist) | 39.1 (chronic tic disorder) and 37.1 (habit disorder) | Tic disorder and habit disorder | RCT | Participants receiving treatment showed significant decrease in tic severity and frequency, and increased control over tics. At 2-year follow-up, 52% of the sample reported 75–100% control over ticcing. |
| O'Connor et al. 2008 | CBT vs. waitlist | $N = 73$ (CBT) vs. 37 (waitlist) | 38.9 (tic disorder), 36.9 (habit disorder), 37.6 (control) | Tic disorder, habit disorder and healthy controls | RCT | CBT was found to significantly improve motor skills compared with waitlist control. Improved motor skills appeared to be related to increased control over tic behaviour, and decreased tic frequency, overactivity and overpreparation in tic disorders. |
| Zarkowska et al. 1989 | RT | 1 | 13 | TS, LD | Single-case study | Tic frequency was reduced during treatment sessions but effects were not maintained outside of sessions. |
| Michultka et al. 1989 | RT | 1 | 19 | TS | Single-case study | Distress, tic frequency and tic intensity were reduced. This was maintained in the 4-week post-treatment phase. |
| Bergin et al. 1998 | RT vs. minimal therapy | $N = 7$ (RT) vs. $n = 9$ (minimal therapy) | 11.8 | TS, ADHD | RCT | At 3-month follow-up, a trend towards improvement in tic expression was noted in both groups, however there were no significant improvements in behavioural measurements. There was no significant difference between the groups. |
| Friedman 1980 | RT and CM | 1 | 11 | TS | Single-case study | Coprolalia improved significantly and motor tics improved slightly but remained problematic. Results were maintained at 4- and 18-month follow-up. |
| Miller 1970 | CM | 1 | 5 | TS | Single-case study | Tics diminished in the home environment where contingency was enforced. CM was then successfully implemented at school. This was maintained at 18-month follow-up. |
| Rosen and Wesner 1973 | CM | 1 | 12 | TS | Single-case study | Tics were almost completely eliminated in a classroom setting where the contingency management was pursued, but remained problematic in the home where the therapy was not used. |
| Schulman 1974 | CM | 1 | 14 | Tic disorder | Single-case study | Tics were eliminated by 11 weeks of treatment but returned post-treatment. |
| Watson and Sterling 1998 | CM | 1 | 4 | Tic disorder | Single-case study | Tic rate was reduced to zero. This was maintained at follow-up. |

Table 1, continued

| Study | Behavioural technique(s) | No. participants | Age range or mean age (years) | Diagnosis | Type of study | Results |
|---|---|---|---|---|---|---|
| Scotti et al. 1994 | CM | 1 | 32 | TS + LD | Single-case study | Escape extinction combined with mild disapproving statements (two items identified as reducing tic expression in the initial assessment) were not found to reduce tic behaviour. |
| Wagaman et al. 1995 | CM | 1 | 9 | Tic disorder | Single-case study | Tic behaviour was decreased to zero. This was maintained at 50-week follow-up. |
| Carr et al. 1996 | CM | 1 | 11 | Tic disorder | Single-case study (observational) | Tics were present in all classroom situations, but were especially high in "escape" situations (negative reinforcement). |
| Roane et al. 2002 | CM | 1 | 22 | Autism and TS | Single-case study | Vocal tics were maintained by automatic reinforcement (being alone in a room with no stimuli). Preference assessment (being given objects depending on the subject's preference) did not reduce tic expression. |
| Himle and Woods 2005 | CM | 7 | 8–12 | TS | Prospective study | Rebound effect occurred in only 1 of 7 participants. |
| Packer 2005 | CM | 71 | 11.1 | TS | Cross-sectional study | Aversive consequences results in worsened tic symptoms, whereas positive reinforcement for modifying a behaviour improved tic symptoms. |
| Himle et al. 2007 | CM | 5 | 8–17 | TS | Single-case studies | 4 of the participants could suppress tics, and 3 of these reported greater premonitory urges than when they were free to tic. |
| Woods and Himle 2004 | CM vs. verbal instructions | 4 | 9-11 | TS | Single-case studies | Verbal instructions produced a 10.3% reduction in tic occurrence compared with DRO (differential reinforcement of zero-rate behaviour) which produced a decrease of 76.3%. |
| Himle et al. 2008 | CM vs. noncontingent reinforcement | 4 | 8–10 | TS, tic disorder | Single-case studies | 3 of the 4 children demonstrated tic suppression in DRO compared to baseline. 1 child demonstrated tic suppression during noncontingent reinforcement. |
| Mansdorf 1986 | AT | 1 | 10 | Tic disorder | Single-case study | 1-year follow-up showed total remission from tic symptoms. |
| Evers and Van de Wetering 1994 | Tension-reduction technique | 2 | 35–40 | Tic disorder | Single-case studies | Tic was eliminated in one case and reduced in the other. These changes were maintained at 3- and 4-month follow-up, respectively. |
| Nagai et al. 2009 | BT | 15 | 29.7 | TS | Prospective study | Biofeedback relaxation training causes a reduction in tic frequency, whereas biofeedback arousal training caused an increase in tic frequency. |

Abbreviations: HRT, habit reversal training; MNP, massed negative practice; SP, supportive psychotherapy; ERP, exposure with response prevention; CBT, cognitive-behavioural therapy; SM, self-monitoring; RT, relaxation therapy; CM, contingency management; AT, assertiveness training; BT, biofeedback training; TS, Tourette syndrome; OCD, obsessive compulsive disorder; ADHD, attention deficit hyperactivity disorder; LD, learning disability; RCT, randomised controlled trial.

### *3.2. Massed negative practice (MNP)*

MNP is based on the notion that over-use of a tic under conditions of massed practice (for instance telling the patient to voluntarily perform a tic for half an hour) leads to the disappearance of that tic through a process called reactive inhibition [42]. Results for the efficacy this method were inconsistent, with some studies reporting a decrease in tic frequency [21,43–49] and others an increase [50–52]. It has been suggested that this variability could depend on patient characteristics, with anxious patients being more susceptible to tic increase in MNP [52]. Overall, MNP appears to be a less effective strategy then HRT for the clinical treatment of TS.

### *3.3. Supportive psychotherapy (SP)*

SP is a conversation-based treatment which focuses on problem solving and reflecting about current life issues [24]. Three studies have evaluated the efficacy of SP versus HRT in the treatment of patients with TS [24–26]. Wilhelm et al. [24,25] found that children and adults HRT groups improved significantly compared to the SP group with regards to both tic severity and functional impairment. Deckersbach et al. [26] confirmed these findings, but also found that SP was as effective as HRT in improving life satisfaction and psychosocial functioning. It has thereby been suggested that SP may be used as an adjunct treatment for individuals with TS, especially for those who do not achieve satisfactory tic reduction with HRT [26]. Overall, the current evidence on SP is inconclusive, especially with regards to the possibility that SP may do no better than a wait-list control, which controls for waxing and waning of symptoms (a comparison that, to the best of our knowledge, has not been conducted yet).

### *3.4. Exposure with response prevention (ERP)*

In ERP, patients are asked to suppress their tics for prolonged periods of time. It is hypothesised that in this way they are exposed to the premonitory sensations (premonitory urges or "sensory tics") associated with tic expression, and begin to habituate to them, thus causing the urge to produce a tic to diminish [53]. Verdellen et al. [54] found that the premonitory urges decreased in severity during ERP, along with a decrease in tic frequency, thus supporting this hypothesis. Verdellen et al. [25] compared the efficacy of ERP to HRT in 43 patients with TS. Both treatments were found to be equally effective in the treatment of tics, however the long-term effects could not be established as 12 participants dropped out from the 3-month follow-up. However, it has to be noted that the ERP group received twice as much therapy time as the HRT condition, which could have explained the lack of a difference in therapeutic effects. Two single-case studies including a total of five patients found the treatment to be effective, although booster sessions were recommended to maintain treatment effects [53,55]. Two studies were carried out testing for the rebound effect post-ERP treatment, whereby tic frequency should increase after an ERP session [56,57]. Neither study demonstrated a rebound effect, thus supporting the theory that patients learn through ERP that the premonitory sensation can be tolerated to some extent, and that there is no need to release tension afterwards [57]. ERP is currently considered to be an efficacious treatment for TS, although long-term effects need to be determined.

### *3.5. Self-monitoring (SM)*

SM requires the patient to acknowledge every tic that he or she produces, for instance by recording it on a counter [35]. This strategy is intended to result in tic reduction through increasing the patient's awareness of the tics he or she is emitting [58]. Eight studies have reported the use of SM in TS. Seven of these were case studies [29,59–64]. Varni et al. [63] used SM in combination with contingency management), whilst Peterson and Azrin [35] compared SM to HRT and relaxation training in 6 patients. Methods sometimes varied between studies. Techniques used included recording tic occurrence on wrist counters or notebooks [60, 64], or acknowledging tic occurrence verbally [63]. Some studies were limited by the fact that they relied of participants to monitor their tics accurately, but some sought the confirmation of external observers (e.g. family members). All of the studies found that SM resulted in a significant reduction of tics, and Peterson and Azrin [35] found the treatment to be somewhat less effective than HRT (44% tic reduction versus 55%). One patient reported an increase in his tics during SM [35]. This exacerbation was apparently due to his attention being constantly drawn to them. In one of the studies, the patient relapsed to original tic frequency at 10-week follow-up [60]. Finally, there is the possibility that increasing awareness of a behaviour and SM are two different processes and that the latter may actually function as a competing response to tics. Overall, SM is potentially an efficacious treatment for TS, but more research is needed to confirm this.

### 3.6. Cognitive-behavioural therapy (CBT)

Four studies have investigated the use of CBT in the treatment of TS and other tic disorders [23,65–67]. All of these studies were carried out by the same Canadian research team. The CBT treatment process followed the principle stages of HRT [19], but in addition addresses the patients' approach to tic situations in a cognitive and behavioural manner, encouraging them to anticipate and appraise high-risk tic situations [65]. A relatively small-sample study ($n = 7$ in each group) found CBT to be as effective as HRT in helping patients to gain control over their tics [23]. Subsequent studies found that CBT was effective in controlling tics [65] and improving skilled motor performance [66], independent of whether or not patients were taking medication for their tics [67]. Although a cognitive approach may not be essential in order to treat tics, it appears to facilitate the process [23].

### 3.7. Relaxation therapy (RT)

RT, where patients are taught to apply a relaxation technique in high-risk tic situations, has been hypothesised to be effective in treating TS, as tics are generally thought to be exacerbated by anger, anxiety and excitement, and are less noticeable in periods of relaxation [68]. The research that has been made into RT as a treatment for TS is mainly limited to single case studies or very small sample studies [35]. Initial results demonstrated improvements in tic symptoms [35,43,46,69,70], however the efficacy of the treatment was often found to be of short duration and patients were prone to relapsing shortly after the therapy ended [51,52,68,71]. The largest and most recent study investigating RT as a treatment for TS, involving a sample of 7 patients in the RT treatment group, concluded that RT is of limited efficacy in the treatment of tics [68].

### 3.8. Assertiveness training (AT)

In the treatment of a disorder like TS, it is important to take into account external as well as internal factors. AT does not focus on the tics themselves but attempts to teach the patient how to respond to their external environment in an assertive manner instead of "resorting" to their tics [46]. Two studies have used this technique in children (one with TS and one with another tic disorder) whose tics were often preceded by an unassertive behaviour [46,72]. In both cases the treatment was found to be effective. These cases highlight the need to take social situations and patients' personalities into account during treatment for TS.

### 3.9. Contingency management (CM)

CM follows the assumption that modifying contextual factors can influence tic expression [73]. A number of studies have investigated the impact that the consequences of producing a tic have on tic expression. Overall, the studies found that positive consequences for withholding a tic decreased tic frequency [63,69,74–81]. Negative consequences such as reprimands tended to increase tic frequency, indicating that drawing attention to a tic reinforces it [60,79,82–84]. A negative reinforcement effect on tics was also demonstrated in a few studies, with increases in tic frequency during escape/avoidance situations [45,82,84,85] and premonitory urge conditions [86]. Many of these studies were carried out in laboratory settings, thus limiting their applicability to real-life situations. In the studies where CM was used as a treatment, long-term effects were found to be variable. In some instances, treatment was deemed to be effective [74,77]. Other studies however found that tics increased to baseline levels when the contingency was not in effect [75,76,84,85]. Himle and Woods [80] investigated the presence of a rebound effect in children told to suppress their tics in a contingency setting, however no rebound effect was found. Because all but one of the studies [79] were single-case studies or had a small sample size, and the methods used were variable, it is difficult to generalise these findings. However it is suggested that in instances where it is possible to modify external factors on a permanent basis (e.g. by instructing parents to reward children for not producing a tic), there is potential for CM in improving the symptoms of TS. It may be more difficult to implement CM in settings other than the patient's home, such as schools.

### 3.10. Tension-reduction

Evers and Van de Wetering [87] suggested a tension-reduction treatment for tics, based on the notion that tics are a tension-reducing response to a specific sensory stimulus. The sensory stimulus had to be identified, and the patient was then taught an alternative response to the stimulus. In this preliminary study, this technique was shown to reduce tic expression in two patients.

### 3.11. Biofeedback training (BT)

BT is a psychophysiological treatment which has shown promise in the management of treatment-resistant epilepsy [88]. In biofeedback, a covert physiological response, such as heart rate, galvanic skin response (GSR), or brain wave pattern, is presented to the participant as online (visual or auditory) feedback, allowing the participant to learn actively to modify the physiological process [89]. Nagai et al. [89] conducted a study investigating how changes in sympathetic arousal affected tic frequency in patients with TS, as a preliminary study to determine the potential for BT as a treatment for TS. Participants attended two sessions of BT, one inducing physiological relaxation, and the other, physiological arousal. Tic frequency was found to be significantly lower during the relaxation session, and higher during the arousal session. It is thus hypothesised that biofeedback relaxation training may potentially be used as a treatment for TS.

## 4. Discussion

This review explored the various existing behavioural treatments that have been used to treat TS. Approaches used were diverse, including techniques to counter tic occurrence, changing patients' outlook on the condition, and even modification of external factors. The review found that out of all the behavioural therapies in the treatment of TS, HRT is by far the best studied. All research papers concluded that HRT is an effective treatment for the disorder, with good results at follow-up [18]. Evidence for the effectiveness of the other treatments is generally limited to a few case-studies and small-sample studies. This is an important limitation in the review as administrations of the same treatment tended to vary between studies, especially CM. ERP and SM appear to be effective treatments for tics, whereas MNP and RT produced contradictory findings suggesting that they were not efficacious. SP, CBT and AT are likely to be found useful as adjunct therapies rather than treatments in their own right. CM has the potential to be effective, provided that it is implemented in a way that suits the individual. A tension-reduction technique tested in two patients showed potential therapeutic benefit. Recently, a study investigated the possibility of using biofeedback training to treat tics.

It is worthwhile considering the use of behavioural treatments in patients with TS, instead of or as well as pharmacotherapy, as they have several advantages. A relatively short course of behavioural therapy has the potential benefit of producing long-lasting remission from tics [90], whereas medications need to be taken indefinitely. Furthermore, drugs carry the potential disadvantages of side effects, non-compliance and unresponsiveness [91]. Behavioural interventions can provide large reductions in tic frequency, not inferior to pharmacological treatment and in some cases bordering total remission [90]. The variable efficacy of behavioural treatments in different patients highlights the need to take individual patient characteristics into account.

As many of the studies conducted were single-case studies, further research is still needed to determine various aspects of the different therapies and their relative efficacy in different patients. Studies are needed for instance to compare the efficacy of the treatments between patients with different severities of TS (e.g., in order of severity, "pure TS" vs. "full-blown TS" vs. "TS-plus") [92], and which behavioural techniques are best suited to each subset of patient. More follow-up studies are required to determine the long-term effects of the treatments. Regression analyses or qualitative studies could help to identify predictors of response and compliance. Finally, the multifaceted clinical phenomenology of TS highlights the need for research using comprehensive outcome measures, including multidimensional disease-specific patient-report measures of health-related quality of life [93].

Although our understanding of the effectiveness of behavioural therapies for TS is largely incomplete, research so far has shown that there is vast scope for the use of these treatments either as alternatives, or adjunct to the traditional pharmacological treatments. From a conceptual point of view, a few questions remain unanswered. For example, how is ERP really different from HRT when patients engage in a competing response and "expose" themselves to the urge or uncomfortable sensation that then goes away after a while? It is essential to pursue research into this area as behavioural techniques may have the potential to make substantial improvement to the health-related quality of life of patients with TS. Future research needs to address better the role of single and multi-component interventions, as some behavioural techniques covered in this review article have become part of multi-component interventions. For example, AT has become an integrative part of CBITS, which was tested in both children [27] and adults [28]. Likewise, CBT interventions in the studies by O'Connor et al. [23,65–67] included HRT and showed results matching with those from studies on HRT [94]. In a way, one of the most relevant questions that remains is not so much whether a particular behavioural technique such as HRT is effective, but whether the additional CBT treatment components added value.

## Acknowledgments

The authors are grateful to Tourettes Action–UK And USA-TSA for their continuing support.

## Conflict of interest

The authors declare that they have no conflict of interest.

## References

[1] American Psychiatric Association. Diagnostic and Statistical Manual of Mental Disorders, 4th Edition, Text Revision (DSM-IV-TR). Washington DC: American Psychiatric Association, 2000.
[2] Robertson MM, Cavanna AE. Tourette syndrome: The facts. Oxford: Oxford University Press (2nd Ed), 2008.
[3] Robertson MM. The prevalence and epidemiology of Gilles de la Tourette syndrome. Part 1: The epidemiological and prevalence studies. J Psychosom Res 2008; 65: 461-472.
[4] Jankovic J. Phenomenology and classification of tics. In: Neurologic Clinics. Jankovic J (Ed). WH Saunders Company, Philadelphia, 1997, pp. 267–275.
[5] Cavanna AE, Critchley HD, Orth M, Stern JS, Young M-B, Robertson MM. Dissecting the Gilles de la Tourette spectrum: A factor analytic study on 639 patients. J Neurol Neurosurg Psychiatry 2011; 82: 1320-1323.
[6] Lombroso PJ, Scahill L. Tourette syndrome and obsessive-compulsive disorder. Brain Dev 2008; 30: 231-237.
[7] Robertson MM. Attention deficit hyperactivity disorder, tics and Tourette's syndrome: The relationship and treatment implications. Eur Child Adolesc Psychiatry 2006; 15: 1-11.
[8] Robertson MM. Mood disorders and Gilles de la Tourette's syndrome: An update on prevalence, etiology, comorbidity, clinical associations, and implications. J Psychosom Res 2006; 61: 349-358.
[9] Frank M, Piedad J, Rickards H, Cavanna AE. The role of impulse control disorders in Tourette syndrome: An exploratory study. J Neurol Sci 2011; 310: 276-278.
[10] Cavanna AE, Robertson MM, Critchley HD. Schizotypal personality traits in Gilles de la Tourette syndrome. Acta Neurol Scand 2007; 116: 385-391.
[11] Singer HS. Tourette's syndrome: From behavior to biology. Lancet Neurol 2005; 4: 149-159.
[12] Frey KA, Albin RL Neuroimaging of Tourette's syndrome. J Child Neurol 2006; 21: 672-677.
[13] Himle MB, Woods DW, Piacentini JC, Walkup JT. Brief review of habit reversal training for Tourette syndrome. J Child Neurol 2006; 21: 719-725.
[14] Robertson MM. Tourette syndrome, associated conditions and the complexities of treatment. Brain 2000; 123: 425-462.
[15] Silva RR, Munoz DM, Barickman J, Friedhoff AJ. Environmental factors and related fluctuation of symptoms in children and adolescents with Tourette's disorder. J Child Psychol Psychiatry 1995; 36: 305-312.
[16] Döpfner M, Rothenberger A. Behavior therapy in tic-disorders with co-existing ADHD. Eur Child Adolesc Psychiatry 2007(Suppl 1) 16:I/89–I/99. doi: 10.007/s00787-007-1011-7.
[17] Moher D, Liberati A, Tetzlaff J, Altman DG, The PRISMA Group. Preferred Reporting Items for Systematic Reviews and Meta-Analyses: The PRISMA Statement. PLoS Med 2009; 6(7): e1000097.
[18] Carr JE, Chong IM. Habit reversal treatment of tic disorders: A methodological critique of the literature. Behav Modif 2005; 29: 858-875.
[19] Azrin NH, Nunn RG. Habit reversal: A method of eliminating nervous habits and tics. Behav Res Ther 1973; 11: 619-628.
[20] Woods DW, Miltenberger RG. Habit reversal: A review of applications and variations. J Behav Ther Exp Psychiatry 1995; 26: 123-131.
[21] Azrin NH, Nunn RG, Frantz SE. Habit reversal vs. negative practice: Treatment of nervous tics. Behav Ther 1980; 11: 169-178.
[22] Miltenberger RG, Fuqua RW, McKinley T. Habit reversal with muscle tics: replication and component analysis. Behav Ther 1985; 16: 39-50.
[23] O'Connor K, Gareau D, Borgeat F. A comparison of a behavioural and a cognitive-behavioural approach to the management of chronic tic disorders. Clin Psychol Psychother 1997; 4: 105-117.
[24] Wilhelm S, Deckersbach T, Coffey BJ, Bohne A, Peterson AL, Baer L. Habit reversal versus supportive psychotherapy for Tourette's disorder: A randomized controlled trial. Am J Psychiatry 2003; 160: 1175-1177.
[25] Verdellen CW, Keijsers GP, Cath DC, Hoogduin CA. Exposure with response prevention versus habit reversal in Tourette's syndrome: A controlled study. Behav Res Ther 2004; 42 (5): 501-511.
[26] Deckersbach T, Rauch S, Buhlmann U, Wilhelm S. Habit reversal versus supportive psychotherapy in Tourette's disorder: a randomized control trial and predictors of treatment response. Behav Res Ther 2006; 44(8): 1079-1090.
[27] Piacentini J, Woods DW, Scahill L, Wilhelm S, Peterson AL, Chang S, Ginsburg GS, Deckersbach T, Dziura J, Levi-Pearl S, Walkup JT. Behavior therapy for children with Tourette disorder: A randomized controlled trial. JAMA 2010; 303: 1929-1937.
[28] Wilhelm S, Peterson AL, Piacentini J, Woods DW, Deckersbach T, Sukhodolsky DG, Chang S, Liu H, Dziura J, Walkup JT, Scahill L. Randomized trial of behaviour therapy for adults with Tourette syndrome. Arch Gen Psychiatry 2012; 69: 795-803.
[29] Ollendick TH. Self-monitoring and self-administered overcorrection: the modification of nervous tics in children. Behav Modif 1981; 5: 75-84.
[30] Finney JW, Rapoff MA, Hall CL, Christophersen ER. Replication and social validation of habit reversal treatment for tics. Behav Ther 1983; 14: 116-126.
[31] Azrin NH, Peterson AL. Habit reversal for the treatment of Tourette syndrome. Behav Res Ther 1988; 26: 347-351.
[32] Azrin NH, Peterson AL. Reduction of an eye tic by controlled blinking. Behav Ther 1989; 20: 467-473.
[33] Sharenow EL, Fuqua RW, Miltenberger RG. The treatment of muscle tics with dissimilar competing response practice. J Appl Behav Analysis 1989; 22: 35-42.
[34] Azrin NH, Peterson AL. Treatment of Tourette syndrome by habit reversal: A waiting-list control group comparison. Behav Ther 1990; 21: 305-318.
[35] Peterson AL, Azrin NH. An evaluation of behavioural treatments for Tourette syndrome. Behav Res Ther 1992; 30: 167-174.
[36] Carr JE, Bailey JS. A brief behaviour therapy protocol for

Tourette syndrome. J Behav Ther Exp Psychiatry 1996; 27: 33-40.
[37] Carr JE, Bailey JS, Carr CA, Coggin AM. The role of independent variable integrity in the behavioural management of Tourette syndrome. Behav Interv 1996; 11: 35-45.
[38] Woods DW, Miltenberger RG, Lumley VA. Sequential application of major habit reversal components to treat motor tics in children. J Appl Behav Anal 1996; 29: 483-493.
[39] Clarke MA, Bray MA, Kehle TJ, Truscott SD. A school-based intervention designed to reduce the frequency of tics in children with Tourette's syndrome. School Psych Rev 2001; 30: 11-22.
[40] Woods DW, Twohig MP. Using habit reversal to treat chronic vocal tic disorder in children. Behav Intervent 2002; 17: 1-10.
[41] Woods DW, Twohig MP, Flessner C, Roloff T (2003) Treatment of vocal tics in children with Tourette syndrome: Investigating the efficacy of habit reversal. J Appl Behav Anal 36: 109-112.
[42] Yates AJ. The application of learning theory to the treatment of tics. J Abnorm Psychol 1958; 56(2): 175-182.
[43] Frederick CJ. Treatment of a tic by systematic desensitization and massed response evocation. J Behav Ther Ex Psychiatry 1971; 2: 281-283.
[44] Nicassio FJ, Liberman RP, Patterson RL, Ramirez E, Sanders N. The treatment of tics by negative practice. J Behav Ther Exp Psychiatry 1972; 3: 281-287.
[45] Lahey BB, McNees MP, McNees MC. Control of an obscene "verbal tic" through timeout in an elementary school classroom. J Appl Behav Anal 1973; 6(1): 101-104.
[46] Tophoff M. Massed practice, relaxation and assertion training in the treatment of Gilles de la Tourette's syndrome. J Behav Ther Exp Psychiatry 1973; 4: 71-73.
[47] Knepler KN, Sewall S. Negative practice paired with smelling salts in the treatment of a tic. J Behav Ther Exp Psychiatry 1974; 5: 189-192.
[48] St James-Roberts N, Powell GE. A case-study comparing the effects of relaxation and massed practice upon tic frequency. Behav Res Ther 1979; 17: 401-403.
[49] Turpin G, Powell GE. Effects of massed practice and cue-controlled relaxation on tic frequency in Gilles de la Tourette's syndrome. Behav Res Ther 1984; 22: 165-178.
[50] Feldman RB, Werry JS. An unsuccessful attempt to treat a tiqueur by massed practice. Behav Res Ther 1966; 4(2): 111-117.
[51] Canavan AGM, Powell GE. The efficacy of several treatments of Gilles de la Tourette's syndrome as assessed in a single case. Behav Res Ther 1981; 19: 549-556.
[52] Crawley B, Powell G. A comparison of the effects of massed practice and relaxation upon the frequency of a facial tic. Behav Psychother 1986; 14: 249-257.
[53] Hoogduin K, Verdellen C, Cath D. Exposure and response prevention in treatment of Gilles de la Tourette's syndrome: Four case studies. Clin Psychol Psychother 1997; 4(2): 125-137.
[54] Verdellen CWJ, Hoogduin CAL, Kato BS, Keijsers GPJ, Cath DC, Hoijtink HB. Habituation of premonitory sensations during exposure and response prevention treatment in Tourette's syndrome. Behav Modif 2008; 32(2): 215-227.
[55] Wetterneck CT, Woods DW. An evaluation of the effectiveness of exposure and response prevention on repetitive behaviors associated with Tourette's syndrome. J Appl Behav Anal 2006; 39: 441-444.
[56] Meidinger AL, Miltenberger RG, Himle M, Omvig M, Trainor C, Crosby R. An investigation of tic suppression and the rebound effect in Tourette's disorder. Behav Modif 2005; 29: 716-745.
[57] Verdellen CW, Hoogduin CA, Keijsers GP. Tic suppression in the treatment of Tourette's syndrome with exposure therapy: The rebound phenomenon reconsidered. Mov Disord 2007; 22: 1601-1606.
[58] Shapiro ES, Cole C. Behavior change in the classroom: Self-management interventions. Guilford Press, New York, 1994.
[59] Thomas EJ, Abrams KS, Johnson JB. Self-monitoring and reciprocal inhibition in the modification of multiple tics of Gilles de la Tourette's syndrome. J Behav Exp Ther Exp Psychiatry 1971; 2: 159-171.
[60] Savicki V, Carlin AS. Behavioral treatment of Gilles de la Tourette syndrome. Int J Child Psychother 1972; 1: 97-109.
[61] Hutzell RR, Platzek D, Logue PE. Control of symptoms of Gilles de la Tourette's syndrome by self-monitoring. J Behav Ther Exp Psychiatry 1974; 5: 71-76.
[62] Billings A. Self-monitoring in the treatment of tics: A single-subject analysis. J Behav Ther Exp Psychiatry 1978; 9: 339-342.
[63] Varni JW, Boyd EF, Cataldo MF. Self-monitoring, external reinforcement, and timeout procedures in the control of high rate tic behaviors in a hyperactive child. J Behav Ther Exp Psychiatry 1978; 9: 353-358.
[64] Wright KM, Miltenberger RG. Awareness training in the treatment of head and facial tics. J Behav Ther Exp Psychiatry 1987; 18: 269-274.
[65] O'Connor KP, Brault M, Robillard S, Loiselle J, Borgeat F, Stip E. Evaluation of a cognitive-behavioural program for the management of chronic tic and habit disorders. Behav Res Ther 2001; 39: 667-681.
[66] O'Connor KP, Lavoie ME, Stip E, Borgeat F, Laverdure A. Cognitive-behaviour therapy and skilled motor performance in adults with chronic tic disorder. Neuropsychol Rehabil 2008; 18(1): 45-64.
[67] O'Connor KP, Laverdure A, Taillon A, Stip E, Borgeat F, Lavoie M. Cognitive behavioural management of tourette's syndrome and chronic tic disorder in medicated and unmedicated samples. Behav Res Ther 2009; 47: 1090-1095.
[68] Bergin A, Waranch HR, Brown J, Carson K, Singer HS. Relaxation therapy in Tourette syndrome: a pilot study. Pediatr Neurol 1998; 18: 136-142.
[69] Friedman S. Self-control in the treatment of Gilles de la Tourette's syndrome: Case study with 18-months follow-up. J Consult Clin Psychol 1980; 48: 400-402.
[70] Michultka DM, Blanchard EB, Rosenblum EL. Stress management and Gilles de la Tourette's syndrome. Biofeedback Self-Regul 1989; 14: 115-123.
[71] Zarkowska E, Crawley B, Locke J. A behavioural intervention for Gilles de la Tourette syndrome in a severely mentally handicapped girl. J Ment Defic Res 1989; 33: 245-253.
[72] Mansdorf IJ. Assertiveness training in the treatment of a child's tics. J Behav Ther Exp Psychiatry 1986; 17(1): 29-32.
[73] Conelea CA, Woods DW. The influence of contextual factors on tic expression in Tourette's syndrome: A review. J Psychosom Res 2008; 65: 487-496.
[74] Miller AL. Treatment of a child with Gilles de la Tourette's syndrome using behavior modification techniques. J Behav Ther Exp Psychiatry 1970; 1: 319-321.
[75] Rosen M, Wesner C. A behavioural approach to Tourette's syndrome. J Consult Clin Psychol 1973; 41: 308-312.
[76] Schulman M. Control of tics by maternal reinforcement. J Behav Ther Exp Psychiatry 1974; 5: 95-96.
[77] Wagaman JR, Miltenberger RG, Williams DE. Treatment of a vocal tic by differential reinforcement. J Behav Ther Exp Psychiatry 1995; 26: 35-39.

[78] Woods DW, Himle MB. Creating tic suppression: Comparing the effects of verbal instruction to differential reinforcement. J Appl Behav Analysis 2004; 34(3): 353-356.

[79] Packer LE. Tic-related school problems: Impact on functioning, accommodations, and interventions. Behav Modif 2005; 29: 876-899.

[80] Himle MB, Woods DW. An experimental evaluation of tic suppression and the tic rebound effect. Behav Res Ther 2005; 43: 1443-1451.

[81] Himle MB, Woods DW, Bunaciu L. Evaluating the role of contingency in differentially reinforced tic suppression. J Appl Behav Analysis 2008; 41(2): 285-289.

[82] Carr JE, Taylor CC, Wallander RJ, Reiss ML. A functional-analytic approach to the diagnosis of a transient tic disorder. J Behav Ther Exp Psychiatry 1996; 27: 291-297.

[83] Watson TS, Sterling HE. Brief functional analysis and treatment of a vocal tic. J Appl Behav Analysis 1998; 31: 471-474.

[84] Roane HS, Piazza CC, Cercone JJ, Grados M. Assessment and treatment of vocal tics associated with Tourette's syndrome. Behav Modif 2002; 26: 482-498.

[85] Scotti JR, Schulman DE, Hojnacki RM. Functional analysis and unsuccessful treatment of Tourette's syndrome in a man with profound mental retardation. Behav Ther 1994; 25: 721-738.

[86] Himle MB, Woods DW, Conelea CA, Bauer CC, Rice KA. Investigating the effects of tic suppression on premonitory urge ratings in children and adolescents with Tourette's syndrome. Behav Rest Ther 2007; 45: 2964-2976.

[87] Evers RAF, Van de Wetering BJM. A treatment model for motor tics based on a specific tension-reduction technique. J Behav Ther Exp Psychiatry 1994; 25: 255-260

[88] Nagai Y, Goldstein LH, Fenwick PBC, Trimble MR. Clinical efficacy of biofeedback treatment on reducing seizures in adult epilepsy: A preliminary randomized controlled study. Epilepsy Behav 2004; 5: 216-223.

[89] Nagai Y, Cavanna A, Critchley HD. Influence of sympathetic autonomic arousal on tics: Implications for a therapeutic behavioural intervention for Tourette syndrome. J Psychosom Res 2009; 67: 599-605.

[90] Peterson AL, Azrin NH. Behavioral and pharmacological treatments for Tourette syndrome: A review. Appl Prev Psychol 1993; 2(4): 231-242.

[91] Cook CR, Blacher J. Evidence-based psychosocial treatments for tic disorders. Clin Psychol Sci Prac 2007; 14: 252-267.

[92] Cavanna AE, Servo S, Monaco F, Robertson MM. The behavioural spectrum of Gilles de la Tourette syndrome. J Neuropsychiatry Clin Neurosci 2009; 21: 13-23.

[93] Cavanna AE, Schrag A, Morley D, Orth M, Robertson MM, Joyce E, Critchley HD, Selai C. The Gilles de la Tourette syndrome-Quality of Life scale (GTS-QOL): Development and validation. Neurology 2008; 71: 1410-1416.

[94] Bate KS, Malouff JM, Thorsteinsson, Bhullar N. The efficacy of habit reversal therapy for tics, habit disorders, and stuttering: A meta-analytic review. Clin Psychol Rev 2011; 31: 865-871.
